# Redetermination of ruizite, Ca_2_Mn^3+^_2_[Si_4_O_11_(OH)_2_](OH)_2_·2H_2_O

**DOI:** 10.1107/S2056989016009129

**Published:** 2016-06-14

**Authors:** Kim V. Fendrich, Robert T. Downs, Marcus J. Origlieri

**Affiliations:** a University of Arizona, 1040 E. 4th Street, Tucson, AZ 85721, USA

**Keywords:** crystal structure, redetermination, ruizite, finite silicate chain

## Abstract

The crystal structure of ruizite, ideally Ca_2_Mn^3+^_2_[Si_4_O_11_(OH)_2_](OH)_2_·2H_2_O [dicalcium dimanganese(III) tetra­silicate tetra­hydroxide dihydrate] was first determined in space group *A*2 with an isotropic displacement parameter model (*R* = 5.6%) [Hawthorne (1984 ▸). *Tschermaks Mineral. Petrogr. Mitt.* **33**, 135–146]. A subsequent refinement in space group *C*2/*m* with anisotropic displacement parameters for non-H atoms converged with *R* = 8.4% [Moore *et al.* (1985 ▸). *Am. Mineral.* **70**, 171–181]. The current study reports a redetermination of the ruizite structure by means of single-crystal X-ray diffraction data of a natural sample from the Wessels mine, Kalahari Manganese Field, Northern Cape Province, South Africa. Our data (*R*_1_ = 3.0%) confirm that the space group of ruizite is that of the first study rather than *C*2*/m*. This work improves upon the structure reported by Hawthorne (1984 ▸) in that all non-H atoms were refined with anisotropic displacement parameters and all hydrogen atoms were located. The crystal structure consists of [010] chains of edge-sharing MnO_6_ octa­hedra flanked by finite [Si_4_O_11_(OH)_2_] chains. The Ca^2+^ cations are situated in the cavities of this arrangement and exhibit a coordination number of seven.

## Abstract 2

The crystal structure of ruizite, ideally Ca_2_Mn^3+^_2_[Si_4_O_11_(OH)_2_](OH)_2_·2H_2_O was redetermined based on single-crystal X-ray diffraction data of a natural sample from the Wessels mine, Northern Cape Province, South Africa, in space group *C*2. All non-H atoms were refined with anisotropic displacement parameters and all hydrogen atoms were located, improving upon previous results and yielding a significantly lower *R* factor.

## Mineralogical and crystal-chemical context   

Ruizite from the Christmas mine, Gila County, Arizona, USA, was originally described by Williams & Duggan (1977 ▸) with monoclinic symmetry in the space group *P*2_1_/*c* and unit-cell parameters *a =* 11.95, *b* = 6.17, *c* = 9.03 Å, β = 91.38° based on rotation and Weissenberg photographs. Ideal chemistry was proposed as CaMn(SiO_3_)_2_(OH)·2H_2_O. Wilson & Dunn (1978 ▸) reported a second occurrence of ruizite from the Wessels mine, Kalahari Manganese Field, Northern Cape Province, South Africa, with the same chemical formula as that given by Williams & Duggan (1977 ▸).

To date, ruizite has been found at five different localities (Table 1 ▸): Christmas mine, Gila County, Arizona, USA (Williams & Duggan, 1977 ▸); Wessels mine (Wilson & Dunn, 1978 ▸) and N’Chwaning mines (Moore *et al.*, 1985 ▸) in the Northern Cape Province, South Africa; Cornwall mine, Lebanon County, Pennsylvania, USA (Kearns & Kearns, 2008 ▸); and the Cerchiara mine, Liguria, Italy (Balestra *et al.*, 2009 ▸). It is a product of retrograde metamorphism and oxidation during cooling of calc-silicate rocks formed *via* contact metamorphism of limestones (Williams & Duggan, 1977 ▸). The secondary mineralization of ruizite at the type locality may have occurred during the Cretaceous or sometime thereafter (Peterson & Swanson, 1956 ▸).

The structure of ruizite was first determined by Hawthorne (1984 ▸) on the basis of space group *A*2, in the same setting as reported by Williams & Duggan (1977 ▸), using a crystal from the Wessels mine. The structure refinement yielded an *R* factor of 5.6% for an isotropic displacement model in which positions of three of the four hydrogen atoms were located. Refinement of anisotropic displacement parameters was not successful. The ideal chemical formula was revised to Ca_2_Mn^III^_2_[Si_4_O_11_(OH)_2_](OH)_2_(H_2_O)_2_. Moore *et al.* (1985 ▸) re-examined the ruizite structure using a sample from the N’Chwaning mine and reported space group *C*2/*m*, a cell setting different from that adopted by Hawthorne (1984 ▸). The structure was refined with anisotropic displacement parameters for non-H atoms, yielding an *R* factor of 8.4%; no hydrogen atoms were located. However, most of the resulting displacement ellipsoids were unreasonable or non-positive definite. Moore *et al.* (1985 ▸) presented a disclaimer that ‘the anisotropic thermal parameters for these crystals are more likely manifestations of inter­growths and domain disorder, rather than descriptions of true thermal motions’.

**Figure 1:**
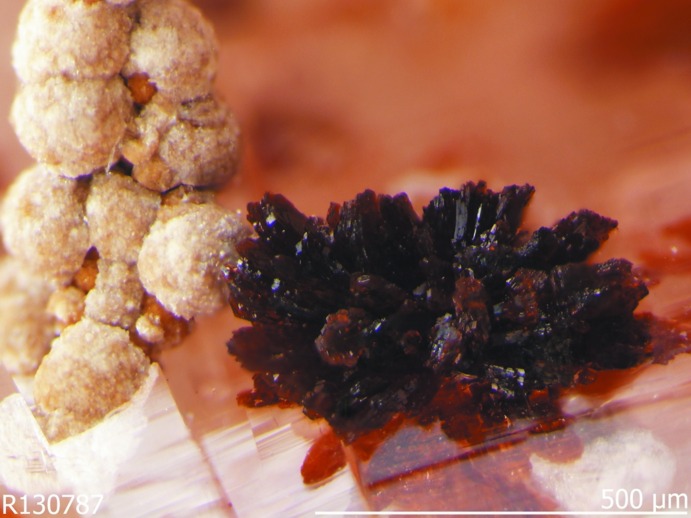
Photograph of the ruizite specimen analyzed in this study.

The current study reports a redetermination of the ruizite structure by means of single-crystal X-ray diffraction data of a natural sample from the Wessels mine, Kalahari Manganese Field, Northern Cape Province, South Africa (Fig. 1 ▸).

## Structural commentary   

**Figure 2:**
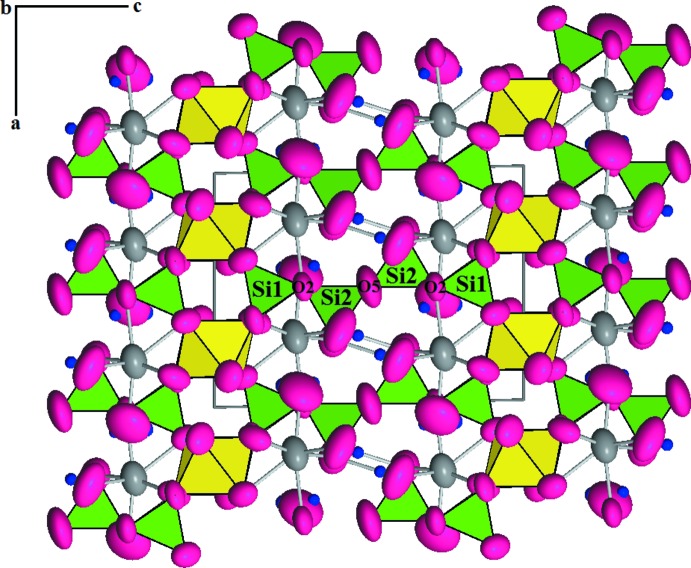
The crystal structure of ruizite as reported in this paper, viewed down *b*. Pink and gray ellipsoids represent O and Ca atoms, respectively. SiO_4_ tetra­hedra are shown in green and MnO_6_ octa­hedra in yellow. Hydrogen atoms are represented by small dark-blue spheres.

The structure of ruizite is characterized by chains of edge-sharing MnO_6_ octa­hedra extending along [010], which are linked by corner-sharing with SiO_4_ tetra­hedra that form short [Si_4_O_11_(OH)_2_] chains, giving rise to a three-dimensional network (Fig. 2 ▸). The finite [Si_4_O_11_(OH)_2_] chain in ruizite is the only reported silicate chain of this type. The relatively large Ca^2+^ cations occupy the inter­stitial cavities and exhibit a coordination number of seven [Ca—O bond-length range 2.348 (4)–2.606 (3) Å]. The Mn^3+^ cations are bonded to four O atoms (O3, O4, and two O1 atoms) and two OH groups (O8—H2), resulting in a distorted MnO_6_ octa­hedron, with an octa­hedral angle variance of 27.35 and a quadratic elongation index of 1.015 (Robinson *et al.*, 1971 ▸). The Si1O_4_ tetra­hedron is more distorted than Si2O_4_, as indicated by angular variances (26.37 *vs* 10.18) and quadratic elongation indices (1.007 *vs* 1.003). Both average Si1—O_nbr_ and Si2—O_nbr_ bond lengths (nbr = non-bridging) are 1.618 Å. The Si2—O7 separation (1.642 Å) is longer than the average Si2—O_nbr_ length because O7 is the hydroxyl group that is also bonded to a Ca^2+^ cation. The O5 atom is located on a twofold rotation axis and bridges the two Si2 atoms with a Si—O bond length of 1.6031 (13) Å, which is most likely a result of the considerably large Si2—O5—Si2 angle [162.9 (3)°] when compared to the Si1—O2—Si2 angle [128.27 (18)°] (Gibbs *et al.*, 1994 ▸).

The hydrogen-bonding scheme in ruizite is presented in Table 2 ▸. The O9 atom is bonded to atoms H3 and H4, forming a water mol­ecule whereas the O7 and O8 atoms are bonded to H1 and H2, respectively, to form two distinct OH groups. The bond-valence calculations (Brown, 2002 ▸) confirm the model (Table 3 ▸). The O6 atom is markedly underbonded because it is an acceptor for both H1 and H3, and consequently it is associated with the shortest Si—O and Ca—O bond lengths. It is inter­esting to note that all hydrogen bonds in ruizite are shorter than 2.85 Å (Table 2 ▸). Nevertheless, the Raman spectrum shows a relatively sharp band at 3570 cm^−1^ (see below). According to Libowitzky (1999 ▸), this band would correspond to a hydrogen bond length (O⋯O) of 3.1–3.3 Å. Perhaps O7—H1 forms a bifurcated hydrogen bond, where H1 is bonded to both O6 and O5. The O7⋯O5 distance is 3.354 Å, which could explain the band at 3570 cm^−1^ (Libowitzky, 1999 ▸).

**Figure 3:**
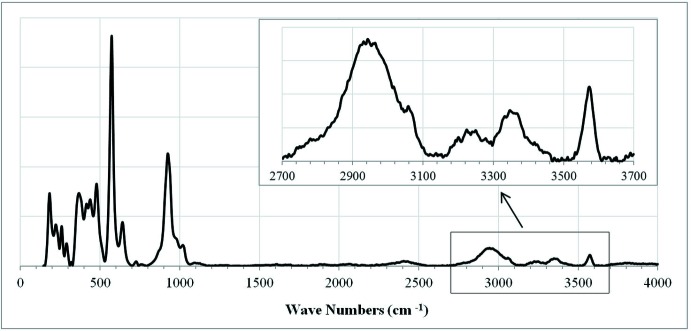
Broad-scan Raman spectrum of an unoriented ruizite specimen (R130787).

Fig. 3 ▸ is a plot of the Raman spectrum of ruizite. A tentative assignment of the major Raman bands is as follows: The bands between 2800 and 3600 cm^−1^ are due to the O—H stretching vibrations. The short H2⋯H4 distance (1.58 Å) may be a result of disordering of one of the hydrogen atoms, which may also explain the considerably broad O—H stretching band in the Raman spectrum around 2940 cm^−1^. The bands in the 1050–800 cm^−1^ region can be attributed to the Si—O stretching vibrations within the SiO_4_ groups and those in the range of 670–520 cm^−1^ to the O—Si—O bending vibrations within the SiO_4_ tetra­hedra. The bands below 500 cm^−1^ are mainly associated with the rotational and translational modes of SiO_4_ tetra­hedra, and the MnO_6_ and CaO_7_ polyhedral inter­actions.

## Synthesis and crystallization   

The ruizite crystal used in this study is from the Wessels mine, Kalahari Manganese Field, Northern Cape Province, South Africa (Fig. 1 ▸) and is in the collection of the RRUFF project (http://rruff.info/R130787). Its chemical composition was measured using a CAMECA SX 100 electron microprobe at the conditions of 15 keV, 20 nA and a beam size <1 µm. The composition, calculated on the basis of 17 oxygen atoms and an estimation of H_2_O by difference is (Ca_1.90_Sr_0.06_Mg_0.04_)(Mn^3+^_1.88_Fe^3+^_0.07_Al_0.05_)Si_4.01_O_11_(OH)_4_·2H_2_O.

The Raman spectrum of ruizite was collected from a randomly oriented crystal at 100% power of 150 mW on a Thermo Almega microRaman system, using a solid-state laser with a wavelength of 532 nm, and a thermoelectrically cooled CCD detector. The laser was partially polarized with 4 cm^−1^ resolution and a spot size of 1 µm.

## Refinement   

Crystal data, data collection and structure refinement details are summarized in Table 4 ▸. The crystal structure was solved and refined based on space group *C*2 because it yielded better refinement statistics than in *C*2/*m* in terms of bond lengths and angles, atomic displacement parameters, and *R* factors. The crystal under investigation was twinned by inversion (Table 4 ▸). Electron microprobe analysis revealed that the ruizite sample studied contains small amounts of Sr, Mg, Fe, and Al. However, the overall effects of minor and trace amounts of these elements are negligible; therefore, the ideal chemical formula Ca_2_Mn^3+^_2_[Si_4_O_11_(OH)_2_](OH)_2_·2H_2_O was assumed during refinement. The H atoms were located from difference Fourier syntheses and confirmed by bond valence sum calculations. Their positions were refined with a fixed isotropic displacement parameter (*U*_iso_ = 0.04). The maximum residual electron density in the difference Fourier map, 0.60 e Å^−3^, was located at 0.85 Å from O8 and the minimum density at 0.17Å from H4.

## Supplementary Material

Crystal structure: contains datablock(s) I. DOI: 10.1107/S2056989016009129/wm5291sup1.cif

Structure factors: contains datablock(s) I. DOI: 10.1107/S2056989016009129/wm5291Isup2.hkl

CCDC reference: 1483820

Additional supporting information: crystallographic information; 3D view; checkCIF report

## Figures and Tables

**Table 1:** Chemical composition and unit-cell parameters (Å, Å^3^) of different ruizite samples

Chemistry	*a*	*b*	*c*	*β*	*V*	Space group	Reference and locality
(Ca_1.90_Sr_0.06_Mg_0.04_)(Mn^3+^_1.88_Fe^3+^_0.07_Al_0.05_)Si_4.01_O_11_(OH)_4_·2H_2_O	9.0360 (3)	6.1683 (2)	11.9601 (4)	91.433 (2)	666.41 (4)	*C*2	Present study R130787, Wessels mine
(Ca_1.96_Mg_0.02_)_Σ=1.98_(Mn^3+^_1.97_Fe^3+^_0.04_Al_0.01_)_Σ=2.02_Si_4_O_11_(OH)_4_·2H_2_O	9.0476 (6)	6.1774 (3)	11.9707 (8)	91.344 (3)	668.9 (1)	*C*2	R140132, Cornwall mine
(Ca_1.98_Mg_0.03_)_Σ=2.01_(Mn^3+^_1.95_Fe^3+^_0.08_V^3+^_0.01_)_Σ=2.04_Si_3.96_O_11_(OH)_4_·2H_2_O	9.056 (5)	6.170 (3)	11.92 (1)	91.30 (4)	666.1 (3)		R060930, Christmas mine
Ca_2_Mn^3+^_2_(OH)_2_[Si_4_O_11_(OH)_2_]·2H_2_O	9.064 (1)	6.171 (2)	11.976 (3)	91.38 (2)	669.7 (4)	*C*2/*m*	Moore *et al.* (1985 ▸), N’Chwaning mine
Ca_2_Mn^3+^_2_[Si_4_O_11_(OH)_2_](OH)_2_(H_2_O)_2_	11.974 (3)	6.175 (2)	9.052 (2)	91.34 (2)	669.1 (4)	*A*2	Hawthorne (1984 ▸), Wessels mine
Ca_1.89_Mn^3+^_2.13_[Si_3.96_O_11_(OH)_2_](OH)_2_·2H_2_O*							Wilson & Dunn (1978 ▸), Wessels mine
Ca_1.06_Mn^3+^_0.86_(SiO_3_)_1.89_(OH)_1.03_·2.06H_2_O	11.95	6.17	9.03	91.38	665.6	*P*2_1_/*c*	Williams & Duggan (1977 ▸), Christmas mine

* Recalculated based on 17 O atoms, as in the currently accepted formula.

**Table 2:** Hydrogen-bond geometry (Å, °)

*D*—H⋯*A*	*D*—H	H⋯*A*	*D*⋯*A*	*D*—H⋯*A*
O7—H1⋯O6^i^	0.73 (6)	1.94 (6)	2.662 (4)	168 (8)
O8—H2⋯O9	0.64 (6)	2.28 (6)	2.842 (4)	149 (9)
O9—H3⋯O6	0.74 (8)	2.06 (8)	2.737 (6)	153 (7)
O9—H4⋯O8	0.72 (6)	2.14 (6)	2.842 (4)	167 (9)

Symmetry code: (i) 
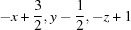
.

**Table 3:** Bond-valence sums

	O1	O2	O3	O4	O5	O6	O7	O8	O9	**Σ_M_**
Ca	0.203	0.178	0.292	0.284		0.357	0.272		0.322	**2.014**
Mn	0.317_×2↓_→		0.674	0.668				0.599_×2_→		**3.173**
Si1	1.044	0.898	0.971	1.036						**3.949**
Si2		0.957			1.058_×2↓_	1.087	0.953			**4.055**
**Σ_O_**	**1.881**	**2.033**	**1.937**	**1.988**	**2.116**	**1.444**	**1.225**	**1.198**	**0.322**	

**Table 4:** Experimental details

Crystal data	
Chemical formula	Ca_2_Mn^3+^_2_[Si_4_O_11_(OH)_2_](OH)_2_·2H_2_O
*M*_r_	582.46
Crystal system, space group	Monoclinic, *C*2
Temperature (K)	293
*a*, *b*, *c* (Å)	9.0360 (3), 6.1683 (2), 11.9601 (4)
β (°)	91.433 (2)
*V* (Å^3^)	666.41 (4)
*Z*	2
Radiation type	Mo *K*α
μ (mm^−1^)	3.13
Crystal size (mm)	0.06 × 0.04 × 0.04

Data collection	
Diffractometer	Bruker APEXII CCD area-detector
Absorption correction	Multi-scan (*SADABS*; Bruker, 2004 ▸)
No. of measured, independent and observed [*I* > 2σ(*I*)] reflections	4997, 2038, 1732
*R*_int_	0.029
(sin θ/λ)_max_ (Å^−1^)	0.758

Refinement	
*R*[*F* ^2^ > 2σ(*F* ^2^)], *wR*(*F* ^2^), *S*	0.030, 0.065, 1.06
No. of reflections	2038
No. of parameters	127
No. of restraints	1
H-atom treatment	Only H-atom coordinates refined
Δρ_max_, Δρ_min_ (e Å^−3^)	0.60, −0.53
Absolute structure	Refined as an inversion twin.
Absolute structure parameter	0.18 (5)

Computer programs: *APEX2* and *SAINT* (Bruker, 2004 ▸), *SHELXT* (Sheldrick, 2015*a* ▸), *SHELXL2014/7* (Sheldrick, 2015*b* ▸), *XtalDraw* (Downs & Hall-Wallace, 2003 ▸) and *publCIF* (Westrip, 2010 ▸).

## supplementary crystallographic information

### Crystal data

**Table:**

Ca_2_H_8_Mn_2_O_17_Si_4_	*F*(000) = 580
*M**_r_* = 582.46	*D*_x_ = 2.903 Mg m^−^^3^
Monoclinic, *C*2	Mo *K*α radiation, λ = 0.71073 Å
*a* = 9.0360 (3) Å	Cell parameters from 1284 reflections
*b* = 6.1683 (2) Å	θ = 8.0–63.1°
*c* = 11.9601 (4) Å	µ = 3.13 mm^−^^1^
β = 91.433 (2)°	*T* = 293 K
*V* = 666.41 (4) Å^3^	Prismatic, brown
*Z* = 2	0.06 × 0.04 × 0.04 mm

### Data collection

**Table:**

Bruker APEXII CCD area-detector diffractometer	1732 reflections with *I* > 2σ(*I*)
Radiation source: fine-focus sealed tube	*R*_int_ = 0.029
φ and ω scan	θ_max_ = 32.6°, θ_min_ = 4.0°
Absorption correction: multi-scan (*SADABS*; Bruker, 2004)	*h* = −13→13
	*k* = −9→8
4997 measured reflections	*l* = −18→18
2038 independent reflections	

### Refinement

**Table:**

Refinement on *F*^2^	Hydrogen site location: difference Fourier map
Least-squares matrix: full	Only H-atom coordinates refined
*R*[*F*^2^ > 2σ(*F*^2^)] = 0.030	*w* = 1/[σ^2^(*F*_o_^2^) + (0.0243*P*)^2^ + 0.6718*P*] where *P* = (*F*_o_^2^ + 2*F*_c_^2^)/3
*wR*(*F*^2^) = 0.065	(Δ/σ)_max_ < 0.001
*S* = 1.06	Δρ_max_ = 0.60 e Å^−^^3^
2038 reflections	Δρ_min_ = −0.53 e Å^−^^3^
127 parameters	Absolute structure: Refined as an inversion twin.
1 restraint	Absolute structure parameter: 0.18 (5)

### Special details

**Table:**

Geometry. All esds (except the esd in the dihedral angle between two l.s. planes) are estimated using the full covariance matrix. The cell esds are taken into account individually in the estimation of esds in distances, angles and torsion angles; correlations between esds in cell parameters are only used when they are defined by crystal symmetry. An approximate (isotropic) treatment of cell esds is used for estimating esds involving l.s. planes.
Refinement. Refined as a 2-component inversion twin.

### Fractional atomic coordinates and isotropic or equivalent isotropic displacement parameters (Å^2^)

**Table:**

	*x*	*y*	*z*	*U*_iso_*/*U*_eq_	
Ca	0.29482 (8)	0.9839 (2)	0.73974 (6)	0.01019 (15)	
Mn	0.74915 (13)	0.7373 (2)	0.99906 (10)	0.00679 (12)	
Si1	0.96440 (10)	0.9873 (3)	0.84877 (8)	0.00607 (18)	
Si2	0.89549 (12)	1.0016 (3)	0.60450 (9)	0.0084 (2)	
O1	0.1260 (3)	0.9855 (9)	0.9082 (2)	0.0088 (5)	
O2	1.0076 (3)	0.9891 (10)	0.71434 (19)	0.0094 (5)	
O3	0.8652 (5)	0.7699 (7)	0.8692 (4)	0.0096 (9)	
O4	0.1302 (4)	0.2031 (7)	0.1276 (3)	0.0070 (9)	
O5	1.0000	1.0402 (7)	0.5000	0.0169 (11)	
O6	0.7769 (4)	1.1913 (6)	0.6146 (3)	0.0121 (8)	
O7	0.8183 (4)	0.7611 (6)	0.5947 (3)	0.0152 (9)	
O8	0.6317 (3)	0.9886 (9)	0.9535 (2)	0.0096 (5)	
O9	0.5570 (4)	0.9743 (11)	0.7213 (3)	0.0201 (8)	
H1	0.792 (8)	0.760 (12)	0.536 (6)	0.040*	
H2	0.617 (7)	1.029 (13)	0.905 (5)	0.040*	
H3	0.594 (7)	1.041 (13)	0.679 (6)	0.040*	
H4	0.586 (6)	0.967 (18)	0.778 (5)	0.040*	

### Atomic displacement parameters (Å^2^)

**Table:**

	*U*^11^	*U*^22^	*U*^33^	*U*^12^	*U*^13^	*U*^23^
Ca	0.0129 (4)	0.0101 (3)	0.0076 (3)	−0.0017 (6)	0.0017 (3)	−0.0006 (6)
Mn	0.0074 (2)	0.0068 (2)	0.0062 (2)	0.00026 (19)	0.00176 (17)	0.00027 (18)
Si1	0.0063 (4)	0.0071 (4)	0.0049 (4)	−0.0002 (8)	0.0009 (3)	0.0002 (8)
Si2	0.0101 (5)	0.0100 (6)	0.0051 (4)	−0.0020 (7)	0.0008 (4)	−0.0003 (7)
O1	0.0071 (11)	0.0098 (10)	0.0094 (12)	0.001 (2)	−0.0006 (9)	−0.002 (2)
O2	0.0094 (12)	0.0142 (11)	0.0045 (11)	−0.001 (2)	0.0012 (9)	0.000 (2)
O3	0.007 (2)	0.010 (2)	0.012 (2)	−0.0012 (16)	0.0025 (18)	−0.0024 (17)
O4	0.009 (2)	0.007 (2)	0.005 (2)	−0.0014 (16)	0.0018 (17)	0.0005 (15)
O5	0.019 (2)	0.025 (3)	0.007 (2)	0.000	0.0043 (17)	0.000
O6	0.0127 (18)	0.0138 (17)	0.0096 (17)	0.0018 (14)	−0.0006 (14)	−0.0011 (13)
O7	0.021 (2)	0.0131 (17)	0.0112 (19)	−0.0035 (16)	−0.0054 (16)	0.0001 (15)
O8	0.0113 (12)	0.0087 (10)	0.0088 (12)	0.001 (2)	−0.0006 (10)	−0.003 (2)
O9	0.0172 (16)	0.0221 (19)	0.0213 (17)	−0.006 (2)	0.0042 (13)	0.001 (3)

### Geometric parameters (Å, º)

**Table:**

Ca—O6^i^	2.348 (4)	Mn—O1^vii^	2.184 (4)
Ca—O9	2.386 (4)	Mn—O1^viii^	2.187 (4)
Ca—O3^ii^	2.422 (5)	Si1—O1^ix^	1.608 (3)
Ca—O4^iii^	2.433 (4)	Si1—O4^x^	1.611 (4)
Ca—O7^ii^	2.449 (4)	Si1—O3	1.635 (5)
Ca—O1	2.557 (2)	Si1—O2	1.664 (2)
Ca—O2^iv^	2.606 (3)	Si2—O6	1.593 (4)
Mn—O3	1.906 (4)	Si2—O5	1.6031 (13)
Mn—O4^v^	1.909 (4)	Si2—O2	1.640 (3)
Mn—O8	1.949 (5)	Si2—O7	1.642 (4)
Mn—O8^vi^	1.951 (5)		
			
O3—Mn—O4^v^	179.1 (3)	O1^vii^—Mn—O1^viii^	179.1 (2)
O3—Mn—O8	89.73 (16)	O1^ix^—Si1—O4^x^	114.1 (2)
O4^v^—Mn—O8	89.97 (16)	O1^ix^—Si1—O3	115.0 (3)
O3—Mn—O8^vi^	90.51 (17)	O4^x^—Si1—O3	110.88 (13)
O4^v^—Mn—O8^vi^	89.81 (16)	O1^ix^—Si1—O2	101.23 (13)
O8—Mn—O8^vi^	179.1 (2)	O4^x^—Si1—O2	107.6 (3)
O3—Mn—O1^vii^	87.33 (17)	O3—Si1—O2	107.0 (3)
O4^v^—Mn—O1^vii^	91.84 (15)	O6—Si2—O5	111.1 (2)
O8—Mn—O1^vii^	99.16 (17)	O6—Si2—O2	112.1 (2)
O8^vi^—Mn—O1^vii^	81.73 (17)	O5—Si2—O2	105.51 (11)
O3—Mn—O1^viii^	92.99 (16)	O6—Si2—O7	112.6 (2)
O4^v^—Mn—O1^viii^	87.85 (16)	O5—Si2—O7	109.7 (2)
O8—Mn—O1^viii^	81.70 (17)	O2—Si2—O7	105.5 (3)
O8^vi^—Mn—O1^viii^	97.41 (16)		

Symmetry codes: (i) *x*−1/2, *y*−1/2, *z*; (ii) *x*−1/2, *y*+1/2, *z*; (iii) −*x*+1/2, *y*+1/2, −*z*+1; (iv) *x*−1, *y*, *z*; (v) *x*+1/2, *y*+1/2, *z*+1; (vi) −*x*+3/2, *y*−1/2, −*z*+2; (vii) *x*+1/2, *y*−1/2, *z*; (viii) −*x*+1, *y*, −*z*+2; (ix) *x*+1, *y*, *z*; (x) −*x*+1, *y*+1, −*z*+1.

### Hydrogen-bond geometry (Å, º)

**Table:**

*D*—H···*A*	*D*—H	H···*A*	*D*···*A*	*D*—H···*A*
O7—H1···O6^xi^	0.73 (6)	1.94 (6)	2.662 (4)	168 (8)
O8—H2···O9	0.64 (6)	2.28 (6)	2.842 (4)	149 (9)
O9—H3···O6	0.74 (8)	2.06 (8)	2.737 (6)	153 (7)
O9—H4···O8	0.72 (6)	2.14 (6)	2.842 (4)	167 (9)

Symmetry code: (xi) −*x*+3/2, *y*−1/2, −*z*+1.
